# Naturally occurring hepatitis B virus surface antigen mutant variants in Malaysian blood donors and vaccinees

**DOI:** 10.1007/s10096-015-2358-1

**Published:** 2015-03-20

**Authors:** S. A. Hudu, N. S. Harmal, M. I. Saeed, A. S. Alshrari, Y. A. Malik, M. T. Niazlin, R. Hassan, Z. Sekawi

**Affiliations:** 1 Department of Medical Microbiology and Parasitology, Faculty of Medicine and Health Sciences, Universiti Putra Malaysia, 43400 UPM Serdang, Selangor Darul Ehsan, Malaysia; 2 Department of Medical Microbiology and Parasitology, Faculty of Basic Medical Sciences, College of Health Sciences, Usmanu Danfodiyo University Sokoto, 840232 Sokoto, Sokoto State Nigeria; 3 Department of Clinical Science, Faculty of Medicine and Health Sciences, Universiti Tunku Abdul Rahman, Kuala Lumpur, Malaysia; 4 Department of Medical Microbiology, Faculty of Medicine and Health Sciences, Sana’a University, Sana’a, Yemen; 5 National Blood Centre Malaysia, Jalan Tun Razak, Kuala Lumpur, 504000 Malaysia

## Abstract

Hepatitis B virus surface mutants are of enormous importance because they are capable of escaping detection by serology and can infect both vaccinated and unvaccinated populations, thus putting the whole population at risk. This study aimed to detect and characterise hepatitis B-escaped mutants among blood donors and vaccinees. One thousand serum samples were collected for this study from blood donors and vaccinees. Hepatitis B surface antigen, antibodies and core antibodies were tested using a commercial enzyme-linked immunosorbent assay (ELISA) kit. DNA detection was performed via nested polymerase chain reaction (PCR), and the *S* gene was sequenced and analysed using bioinformatics. Of the 1,000 samples that were screened, 5.5 % (55/1,000) were found to be HBsAg-negative and anti-HBc- and HBV DNA-positive. All 55 isolates were found to belong to genotype B. Several mutations were found across all the sequences from synonymous and non-synonymous mutations, with the most nucleotide mutations occurring at position 342, where adenine was replaced by guanine, and cytosine at position 46 was replaced by adenine in 96.4 % and 98 % of the isolates, respectively. Mutation at position 16 of the amino acid sequence was found to be common to all the Malaysian isolates, with 85.7 % of the mutations occurring outside the major hydrophilic region. This study revealed a prevalence of 5.5 % for hepatitis B-escaped mutations among blood donors and vaccinated undergraduates, with the most common mutation being found at position 16, where glutamine was substituted with lysine.

## Introduction

Hepatitis B virus (HBV) is the most common chronic viral infection worldwide, affecting about 2 billion people globally, with 378 million chronic carriers [1]. The HBV genome encodes four genes, called *C*, *X*, *P* and *S*. The *C* gene codes for a core protein known as the hepatitis B core antigen (HBcAg), while the *X* gene encodes the X protein, which is the protein that circulates in the blood of infected individuals when there is active viral replication [2]. HBsAg has a long open-reading frame (ORF), which has three in-frame ‘start’ codons (ATG) that split the gene into three sections: PreS1, PreS2 and S. As a result of the numerous start codons, different-sized polypeptides are produced, known as the large (PreS1 + PreS2 + S), medium (PreS2 + S) or small (S) polypeptides [3].

The hepatitis B surface antigen is a spherical particle that measures 22 mm in diameter. Its determinant is a double-loop structure, which projects from the surface of the virion and forms the key neutralising epitope [4]. This determines the surface antigen known as the ‘a’ determinant gene, and is the main target for the vaccine, including antibodies. However, mutation in the surface protein as a result of amino acid deletions or substitutions, particularly in the region of amino acids 137–147, permits hepatitis B virus replication in vaccinated subjects. This is because antibodies induced by the current vaccine may not recognise changes in the surface antigen as a result of transformation (mutation). HBV surface mutants are of enormous importance because they are capable of infecting both vaccinated and unvaccinated individuals, thus putting the whole population at risk. Clinically important hepatitis B mutants have been reported in all genotypes [5], indicating a wide spread across the various genotypes. Therefore, understanding the prevalence of such mutations would be useful for the design of a diagnostic assay and in the prevention and treatment of HBV. However, extensive molecular characterisation of occult hepatitis B strains in Southeast Asia has not been performed. This study provides new information regarding the phylogenetic analysis of vaccine-escaped hepatitis B strains obtained from blood donors and vaccinated undergraduate volunteers in Malaysia.

## Patients and methods

### Sample collection

A total of 1,000 serum samples were collected for this study: 500 samples from blood donors at the National Blood Centre Malaysia in Kuala Lumpur and 500 samples from volunteer undergraduate students of the Faculty of Medicine and Health Sciences, Universiti Putra Malaysia (Table 1).

**Table 1:** Distribution of study populations and their characteristics

Study cohort	No. of samples	HBsAg	Anti-HBc		PCR	
			Positive	Negative	Positive	Negative
Blood donors	500	Negative	35	465	35	465
Undergraduate students	500	Negative	20	480	20	480
Total	1,000	1,000	55	945	55	945

### Serological assay

The National Blood Centre Malaysia performed HBsAg testing using an ABBOTT PRISM instrument (Abbott Laboratories, Abbott Park, IL, USA). Each of the samples was re-tested for HBsAg in the laboratory of Medical and Molecular Virology, Faculty of Medicine and Health Sciences, Universiti Putra Malaysia. Anti-HBs, HBsAg and anti-HBc were tested using a commercial enzyme-linked immunosorbent assay (ELISA) kit (DRG International, Inc., New York, USA), according to the manufacturer’s instructions. Twenty randomly selected samples were sent to a commercial laboratory to verify the result of our ELISA. The sensitivity for the serological assays was 10 IU/L optical density (OD ≥ 0.105), 0.5 ng/mL (OD ≥ 0.105) and 2 ng/mL (OD ≤ 0.600).. Sera with repeatedly positive results were considered positive and those with repeatedly negative results were considered negative for each of the serological markers.

### HBV DNA isolation and detection from serum

HBV DNA was extracted from 200 μl of serum using the QIAamp DNA Blood Mini Kit (Qiagen, Hilden, Germany), according to the manufacturer’s instructions. Briefly, 20 μl of protease was placed into a 1.5-μl tube, and 200 μl of the serum and AL buffer was added to each tube before being vortexed and incubated at 56 °C for 10 min. Then, 200 μl of ethanol was added to enhance DNA precipitation. To eliminate impurities, the trapped DNA was washed in two steps using AW1 and AW2 buffer as the first and second steps, respectively. The DNA was eluted with 50 μl of elution buffer and stored at −20 °C.

Two sets of primers were designed to amplify the *S* gene in a nested polymerase chain reaction (PCR). The first round targeted a 916-bp segment of the envelope gene and the second round of PCR was performed using the internal primers to amplify 656 bp of the *S* gene (Table 2). Samples were handled in aseptic conditions and all PCR reactions were carried out in duplicate. In the first round was performed using 20 μl of reaction mixture containing 0.5 μl DNA sample, 10 μl Maxime PCR PreMix Kit (i-pfu) (iNtRON Biotechnology Inc., Korea), 0.5 μl of each forward and reverse primer, and 8.5 μl nuclease-free water. The second round was performed in a 50-μl reaction mixture containing 1 μl DNA sample, 25 μl Maxime PCR PreMix Kit (i-pfu) (iNtRON Biotechnology Inc., Korea), 1.25 μl of each forward and reverse primer, and 21.5 μl nuclease-free water. The PCR conditions comprised an initial denaturation step of 5 min at 94 °C, 30 cycles of 94 °C for 5 min, 94 °C for 30 s, 63.8 °C for 30 s and 72 °C for 60 s, and a final extension at 72 °C for 8 min; both reactions were performed under the same conditions. The products of amplification were recovered from a 1.5 % (w/v) agarose gel using a DNA purification kit (Invitrogen Corporation, San Diego, CA, USA), according to the manufacturer’s instructions.

**Table 2:** Oligonucleotide primers used to amplify the hepatitis B *S* gene by nested polymerase chain reaction (PCR)

Primers	Sequence (5′–3′)	Product length (bp)
	Outer primer	
HBsAg forward	ACTGTCTCTGCCATATCGTCA	916
HBsAg reverse	AACCCCAAAAGACCCACAA	
	Inner primer	
HBsAg forward	ACATGGAGAACATCGCATCAG	656
HBsAg reverse	AATTGGTAACAGCGGTATAAAGG	

### Sequencing and genotyping

The purified DNA of 55 sera from both blood donors and vaccinated undergraduates was re-amplified using nested PCR as described above, and the products were commercially sequenced (First BASE Laboratories, Selangor, Malaysia). All sequences were submitted to the NCBI GenBank (http://www.ncbi.nlm.nih.gov/genbank) under accession numbers KC953665–KC953699 and KF011208–KF011227 for blood donors and vaccinated undergraduates, respectively (Table 3). The sequences were assembled using BioEdit v7.2.0, after which they were manually edited to remove gaps or ambiguously aligned sites. All sequences were aligned to reference sequences from GenBank using ClustalW via Molecular Evolutionary Genetics Analysis version 5 (MEGA 5) software [6]. Phylogenetic analysis was carried out using the maximum likelihood (ML) method. Reference sequences were selected from the GenBank database. For genotyping, our sequences were aligned with *S* gene sequences from established HBV genotypes A–I from the NCBI GenBank. The accession numbers are as follows: genotype A (AY161138, AJ309371), B (AB0738554, AB073830), C (AF223960, X75656), D (AY161159, AB090270), E (X75657, AB074845), F (AF223963, AB036908), G (AF405706, M74499), H (AY090457, AY090460) and I (FJ023667, AF241407). Evolutionary history was inferred using the ML method, selecting topology with a superior log likelihood rate. A distinct gamma distribution was used to model evolutionary rate differences among sites (+G, parameter = 0.7185). Genotypes were determined based on the genetic distance and clustering of the DNA sequences [7].

**Table 3:** List of sequences submitted to the NCBI GenBank along with their accession numbers

No.	Isolates	Genotype	CDS	Accession number	Country of origin
1	UPMD17	B	1-599	KC953665	Malaysia
2	UPMD47	B	1-598	KC953666	Malaysia
3	UPMD76	B	1-597	KC953667	Malaysia
4	UPMD79	B	1-598	KC953668	Malaysia
5	UPMD95	B	1-596	KC953669	Malaysia
6	UPMD110	B	1-592	KC953670	Malaysia
7	UPMD111	B	1-600	KC953671	Malaysia
8	UPMD124	B	1-594	KC953672	Malaysia
9	UPMD133	B	1-599	KC953673	Malaysia
10	UPMD138	B	1-582	KC953674	Malaysia
11	UPMD141	B	1-597	KC953675	Malaysia
12	UPMD161	B	1-594	KC953676	Malaysia
13	UPMD165	B	1-593	KC953677	Malaysia
14	UPMD16	B	1-599	KC953678	Malaysia
15	UPMD172	B	1-594	KC953679	Malaysia
16	UPMD197	B	1-575	KC953680	Malaysia
17	UPMD228	B	1-598	KC953681	Malaysia
18	UPMD265	B	1-593	KC953682	Malaysia
19	UPMD288	B	1-596	KC953683	Malaysia
20	UPMD290	B	1-595	KC953684	Malaysia
21	UPMD329	B	1-597	KC953685	Malaysia
22	UPMD348	B	1-596	KC953686	Malaysia
23	UPMD349	B	1-595	KC953687	Malaysia
24	UPMD351	B	1-593	KC953688	Malaysia
25	UPMD368	B	1-598	KC953689	Malaysia
26	UPMD375	B	1-593	KC953690	Malaysia
27	UPMD403	B	1-593	KC953691	Malaysia
28	UPMD459	B	1-596	KC953692	Malaysia
29	UPMD469	B	1-595	KC953693	Malaysia
30	UPMD518	B	1-597	KC953694	Malaysia
31	UPMD522	B	1-593	KC953695	Malaysia
32	UPMD525	B	1-593	KC953696	Malaysia
33	UPMD567	B	1-596	KC953697	Malaysia
34	UPMD301	B	1-604	KC953698	Malaysia
35	UPMD170	B	1-583	KC953699	Malaysia
36	UPMS45	B	1-622	KF011208	Malaysia
37	UPMS139	B	1-621	KF011209	Malaysia
38	UPMS140	B	1-622	KF011210	Malaysia
39	UPMS141	B	1-624	KF011211	Malaysia
40	UPMS148	B	1-621	KF011212	Malaysia
41	UPMS176	B	1-626	KF011213	Malaysia
42	UPMS181	B	1-627	KF011214	Malaysia
43	UPMS186	B	1-611	KF011215	Malaysia
44	UPMS189	B	1-611	KF011216	Malaysia
45	UPMS194	B	1-613	KF011217	Malaysia
46	UPMS318	B	1-621	KF011218	Malaysia
47	UPMS339	B	1-614	KF011219	Malaysia
48	UPMS340	B	1-605	KF011220	Malaysia
49	UPMS343	B	1-605	KF011221	Malaysia
50	UPMS379	B	1-625	KF011222	Malaysia
51	UPMS388	B	1-611	KF011223	Malaysia
52	UPMS389	B	1-622	KF011224	Malaysia
53	UPMS402	B	1-621	KF011225	Malaysia
54	UPMS256	B	1-625	KF011226	Malaysia
55	UPMS354	B	1-610	KF011227	Malaysia

## Results

**Fig. 1:**
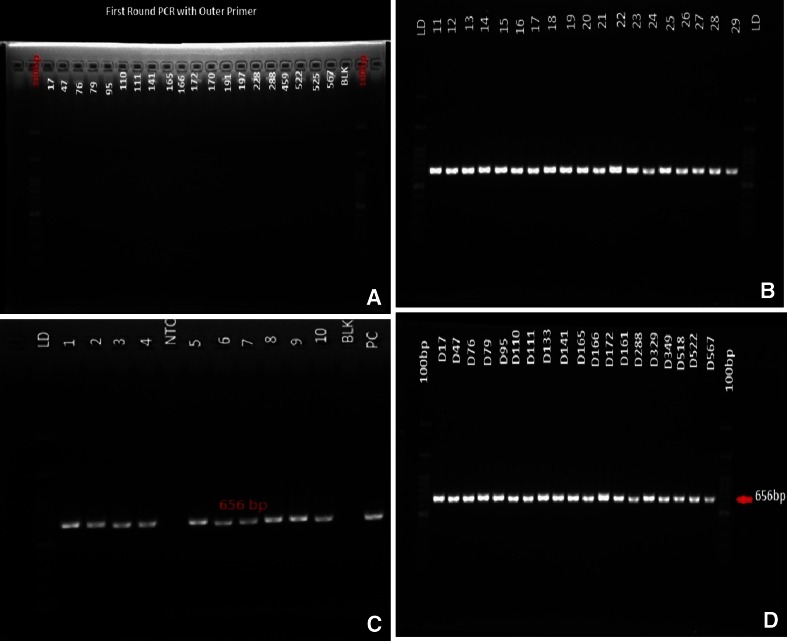
Panel A shows the first-round polymerase chain reaction (PCR) gel; panel B shows the second-round PCR gel from the sample of students; panel C shows the controls and blank used in the experiment; panel D shows the second-round PCR from blood donors. A 656-bp DNA product was amplified using primers specific for the hepatitis B *S* gene. All lanes show a PCR product of the expected size (B, C and D), except lane A, which showed no band in the first round PCR. *LD*: 100-bp molecular weight marker; *PC*: positive control; *NTC*: no-template control

Of the 1,000 samples that were screened, 5.5 % (55/1,000) were found to be HBsAg-negative and anti-HBc-positive, all of which were found to be positive for HBV DNA by nested PCR. Additionally, 84.8 % (848/1,000) of the samples were anti-HBs-positive. The results of the nested PCR showed the presence of a 656-bp band that was specific for the hepatitis B *S* gene fragment, indicating a positive result (Fig 1). Of the 5.5 % (55/1,000) of the samples that were positive, 87.3 % (48/55) were also hepatitis B surface antibody-positive, suggesting immunity as a result of previous infection [8], while 12.7 % (7/55) were anti-HBs-negative. The latter group is referred to as isolated anti-core, as has been previously described [9]. However, none of these samples were hepatitis B surface antigen-positive.

**Fig. 2:**
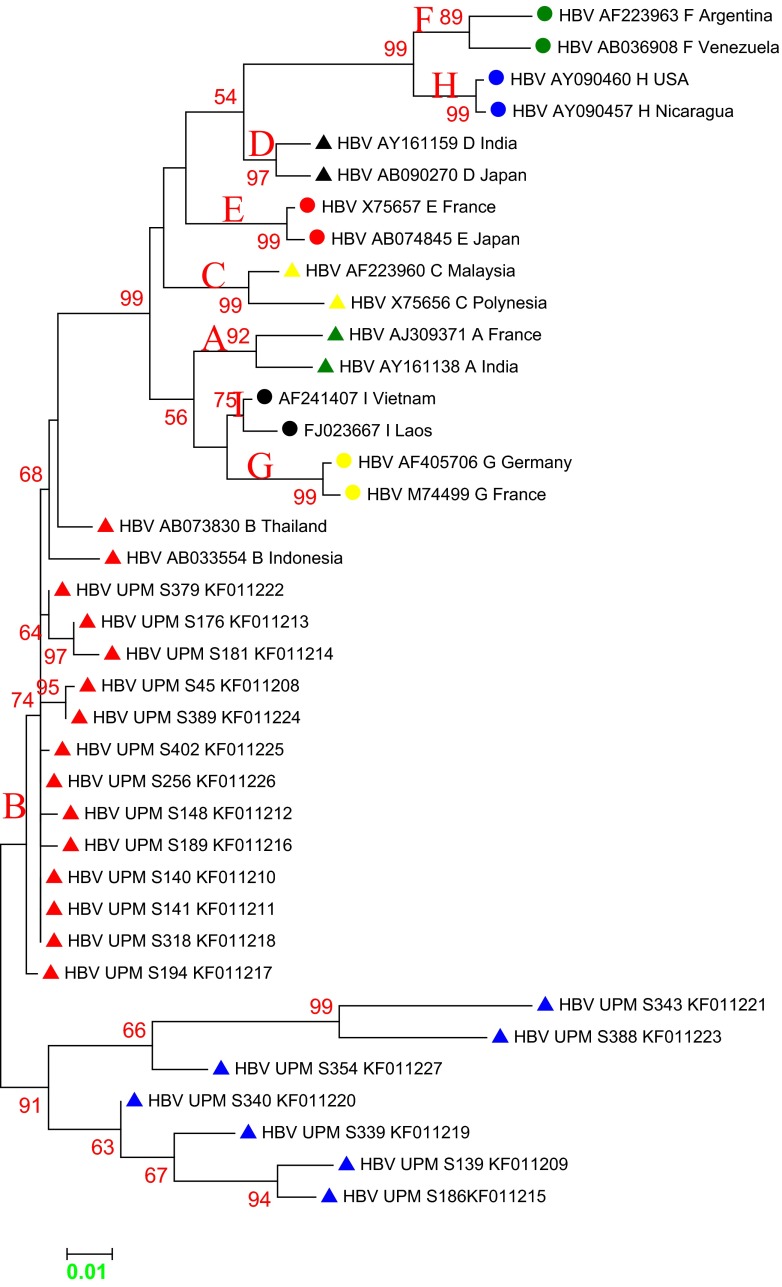
Molecular phylogeny of the *S* gene of 35 Malaysian isolates from vaccinated individuals and reference sequences from established genotypes (A to H) from the NCBI GenBank. The *blue triangles* indicate aberrant strains. The evolutionary relationship was inferred using the maximum likelihood (ML) method based on the Kimura model. Malaysian isolates are indicated by *HBV UPM D* followed by isolate number and all belong to genotype B, with the exception of the aberrant strains

**Fig. 3:**
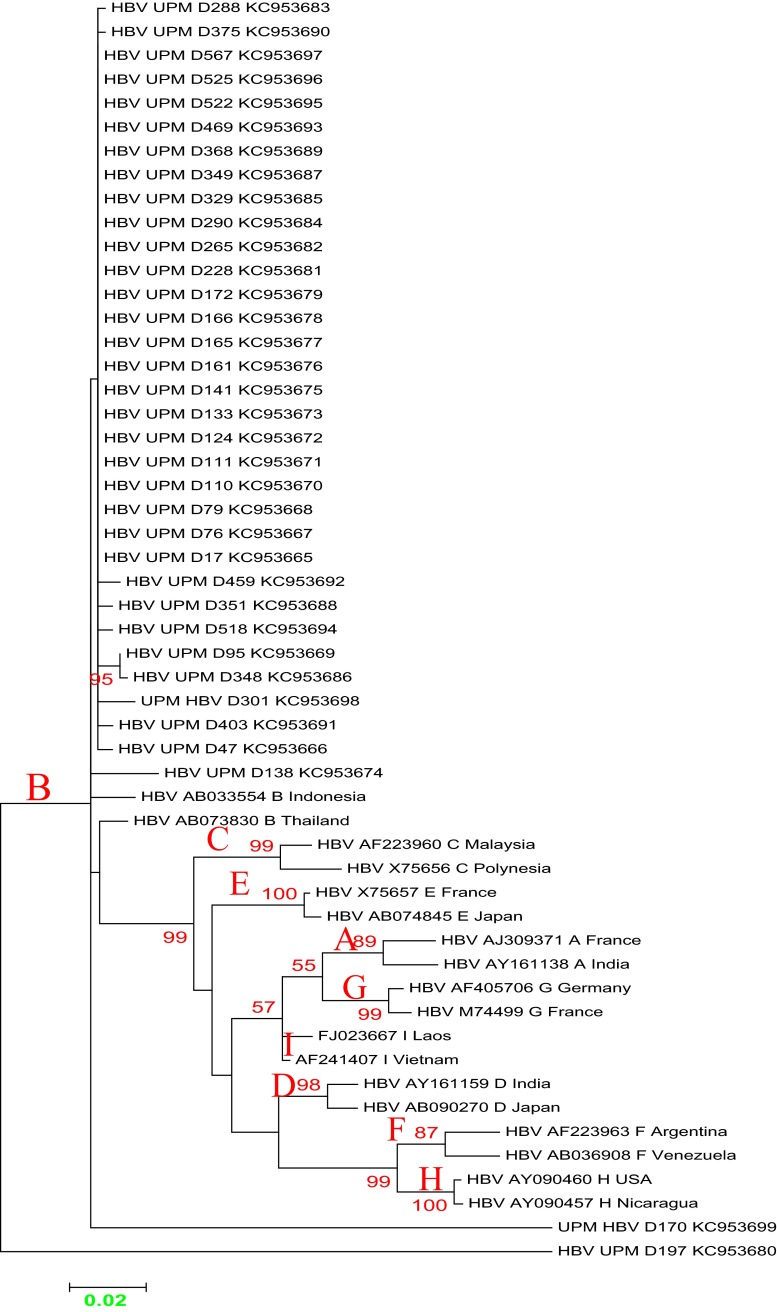
Phylogeny of the *S* gene of 35 Malaysian isolates from blood donors and reference strains from established genotypes (A to H) from the NCBI GenBank. The *blue triangles* indicate an aberrant genotype. The evolutionary relationship was inferred using the ML method based on the Kimura model. Malaysian isolates are indicated by *HBV UPM D* followed by isolate number and all belong to genotype B, with the exception of the aberrant strains

**Fig. 4:**
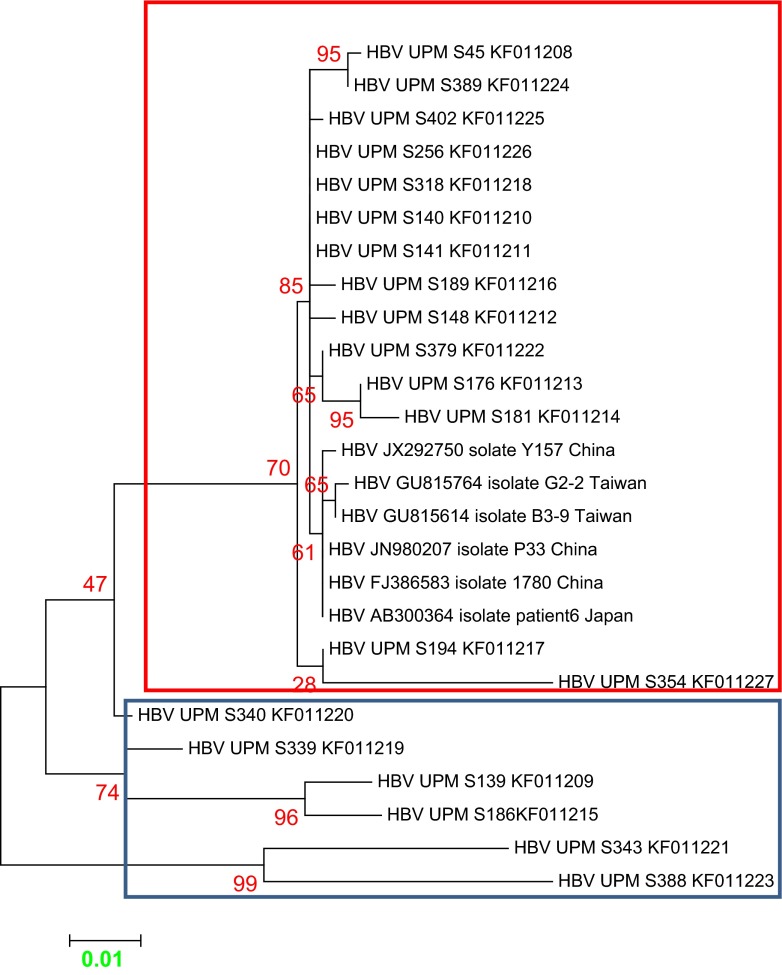
Molecular phylogenetic relationship of Malaysian hepatitis B virus (HBV) isolates from vaccinated individuals with reference isolates from the NCBI GenBank. The evolutionary relationship was inferred using the ML method based on the Jukes–Cantor model. Malaysian isolates are separated into two distinct clusters marked in frames (*red* and *blue*)

Multiple sequence alignment of Malaysia vaccine-escaped hepatitis B strains revealed a sequence homology of 99 % and a sequence identity that ranged from 83 to 100 %, and pairwise distances ranging from 0.000 to 0.177, with an overall mean distance of 0.029. All of our isolates belonged to genotype B, with some aberrant sequences showing a distant relationship (Figs. 2 and 3). The majority of the Malaysian isolates from blood donors fell into the first cluster (blue), and were similar to the Panama isolate B2 and other isolates from Japan, China and Taiwan. Two of our isolates fell within the second cluster (red), together with isolates from Canada, the Philippines and Indonesia. In the third cluster (green), isolate UPMD197 showed a distant relationship with other isolates, but was similar to isolate KC315324 from Thailand. Malaysian isolates from vaccines are phylogenetically related to other isolates from China, Taiwan and Japan, except for isolates UPMS340, UPMS139, UPMS186, UPMS343 and UPMS388, which were distantly related and belong to a different clade and sub-clades (Fig. 4). The most commonly occurring nucleotide mutations were the replacement of adenine at position 342 by guanine (A342G) and cytosine at position 46 by adenine (C46A); these were present in 96.4 % (53/55) and 98 % (54/55) of the isolates, respectively.

**Fig. 5:**
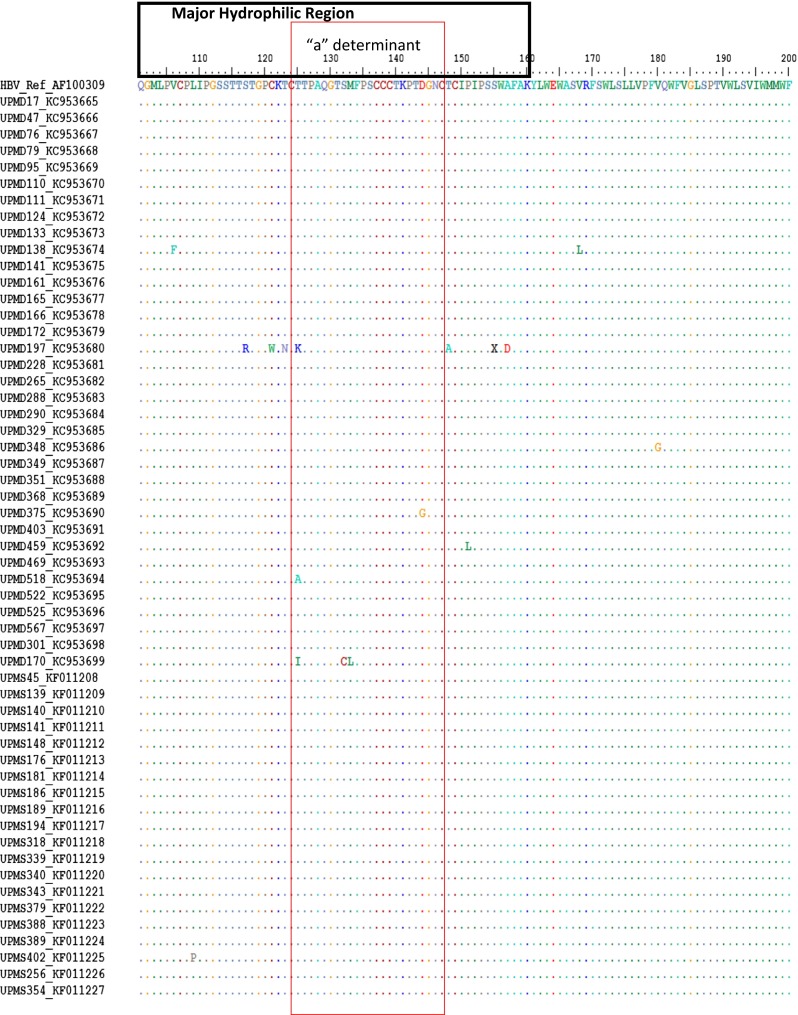
Multiple sequence alignment of deduced amino acids of hepatitis surface antigen major hydrophilic region (MHR) of 55 Malaysian sequences compared with a reference sequence from the NCBI GenBank. The antigenic region (*a* determinant) is boxed (*red*). The MHR is from 100 to 160 aa and the *a* determinant from 124 to 147 aa

Aligned deduced amino acid sequence showed corresponding mutations at various points of the amino acid sequences of the surface protein, including substitutions to deletions. The mutation at amino acid 16 is common to all of the Malaysian isolates, resulting in the substitution of glutamine (Q) with lysine (K) in 53 of the isolates, while the remaining two isolates contained an arginine residue or a stop codon. A total of 105 amino acid mutations were found in all 55 Malaysian isolates; 85.7 % (90/105) were outside the major hydrophilic region (MHR) and 14.3 % (15/105) were within the MHR, of which 40 % (6/150) were within the *a* determinant region (Table 4). The location of amino acid mutations within the MHR (100–160 aa) and the *a* determinant region (124–147 aa) are shown in Fig. 5.

**Table 4:** Distribution of amino acid (aa) mutations within different regions of the surface protein. The *a* determinant region is within the major hydrophilic region (MHR)

*a* Determinant region (124–147 aa)	MHR (100–160 aa)	Outside MHR
T125K	V106F	Q16K, G18V, G18R, G18M, L95Stop
D144G	S117R	T37N, T37I, S61Stop, F19L, F20L, W35R, W35G, W36C, W36R, G50A, G50D, G50V, H60P, H60S, R73L, I82L, V168L
T125A	C121W	T23I, V47G, C48G, C48V, L49I, Q54H
T125I	T123N	I25N, S55P, S55G, L12Q, L12P, L12R
S132C	T148A	V14L, V14G, Q16R, V180G, N52S, D99G
M133L	S155X	P29L, L13F, P11T, Q56T, S58Y, S132T
	A157D	165Stop, L15Stop, I28T, I28E, S59R
	T125K	S59N, S64F, S64G, I68V, C69F, C90F
	D144G	C90R, C90S, F93V, F93L, L94M, I92H
	T125A	I92L, Q51Stop, F80L, C85G, C85F, C85L
	T125I	I86F, I86V, T63S, L88M, T27R, Q30R
	S132C	D33N, D33S, N40D, P62H, P62Q, R78W
	M133L	L89R, W74R, P66L, L39I, L39T, K24Q
	P151L	I57T, S53Y, Q54Stop
	L109P	
Total: 6	15	90

## Discussion

In this study, HBV DNA was detected in HBsAg-negative serum using nested PCR, which is in line with previous studies that demonstrated the same result in peripheral blood mononuclear cells, serum and liver samples that were negative for HBsAg, HBV DNA levels were less than 10^4^ copies/mL [10], which is considerably lower than those that are HBsAg-positive. Our study revealed a 5.5 % prevalence of the hepatitis B core antibody among blood donors and vaccinated undergraduate students, which is similar to that reported in Italy [11], Egypt [12], Germany, the UK [13] and the USA [14], but less than that reported in Korea [15] and Greece [16]. This study revealed a 100 % prevalence of vaccine-escaped hepatitis among hepatitis B core antibody-positive blood donors and vaccinated undergraduates. This is similar to the frequency reported in China [17]. Additionally, other studies have reported the identification of vaccine-escaped hepatitis B virus more frequently in individuals with anti-HBc-positive serology than in those with anti-HBc-negative serology, which is in accordance with our findings. On the other hand, a prevalence of 33.3 % was reported in northeast China [17], compared to 0.11 % in Taiwan [18]. Occult or vaccine-escaped hepatitis B infections have significant clinical importance, since they can become reactivated when the immune system is suppressed and can be transmitted through the transfusion of blood or blood products, organ transplant and sexual intercourse. It may also enhance the progression of liver fibrosis and, subsequently, hepatocellular carcinoma.

Our results showed that all isolates belonged to a single genotype, B, which is in agreement with previous studies that confirmed genotypes B and C as the most prevalent in Asia [19]. However, in Malaysia, genotype B was reported to be dominant over C [20], which is in contrast to the findings in asymptomatic carriers in China, where genotype C was reported as being dominant [5].

Most of the sequences were found to exhibit a close relationship with reference isolate 14 (JX869999) from Panama, which belongs to genotype B, serotype *adw* ^2^ and was isolated from plasma. Other isolates, including UPMD301 and UPMD170, were closely related to reference isolates from Canada, the Philippines and Indonesia. One of the Malaysian isolates, UPMD179, was found to be distantly related to all other isolates, but to have a close relationship with isolate M13008 (KC315324) from Thailand, which belong to genotype B, serotype *adw*, isolated from serum [21]. On the other hand, sequences from vaccinated Malaysian individuals were separated into two clusters, the majority of which were found to have a close evolutionary relationship with reference isolates from China, Taiwan and Japan. However, some sequences from vaccinated individuals were distantly related to other sequences belonging to different clades and sub-clades as a result of frequent mutations within the *S* gene.

The hepatitis B viral DNA polymerase enzyme lacks proofreading ability, making it highly susceptible to nucleotide misincorporation during reverse transcription or replication [22]. Mutations in the *S* gene are the most concerning from a clinical point of view, because of the difficulty in establishing a diagnosis, leading to the progression of undiagnosed cases to occult chronic infection, liver failure and, subsequently, hepatocellular carcinoma. The development of mutations in the *S* gene of HBV interferes with the viral antigen recognition by antibodies in serological assays, as well as with the response to recombinant hepatitis B vaccine. Several types of mutations were implicated, many of which affect amino acids within the *a* determinant region of the *S* gene; however, our results show that the majority (94.3 %) of mutations occurred outside the major hydrophilic region. As all of our sequences have mutations outside the *a* determinant region, it can be concluded from our findings that mutations outside the *a* determinant region may play a significant role in vaccine-escaped hepatitis B mutant infection as a result of the failure to detect HBsAg in the sera. Only 6/105 (5.7 %) of the mutations were located within the *a* determinant region: T125K, D144G, T125A, S132C and M133L. Therefore, our findings suggest that mutations within the *a* determinant region, as well as other regions of the *S* gene, may play a significant role in the diagnosis and detection of vaccine-escaped hepatitis B infection, which is in agreement with the findings from previous studies. However, we found mutations at positions 133 and 144 in isolates UPMD170 and UPMD375, respectively, which concurred with reports from China and Turkey [23] and were described as ‘immune-selected mutations’ [24]. However, the mutation at position 133 aa creates an N-linked glycosylation site, which can effectively rescue the effects of the G145R mutant virion and, to some extent, other immune-escaped mutants [25]. Premature stop codons were found in isolates UPMD47, UPMD95, UPMD348, UPMS45, UPMS139, UPMS186, UPMS388 and UPMS389, which might affect HBsAg production, as described previously [26].

The most prevalent mutation found in this Malaysian isolate was the substitution of glutamine at position 16, which is in contrast to the previous report of G145R being the most common mutation [27]. None of our isolates contained a mutation at position 145 of the amino acid sequence and, yet, HBsAg could not be detected using commercial ELISA kits. Thus, it can be speculated that mutants with defects in the *S* gene of the HBV genome at position 16 lead to the loss of HBsAg expression, which is the main target for serological diagnosis. This might be the result of post-transcriptional effects of mutations on HBsAg expression, as described previously [28], resulting in HBV-escaped mutant infection; however, further functional studies in cell culture are required. This suggests that mutations outside the MHR can also lead to the development of escaped mutant strains of hepatitis B and subsequent serological diagnostic failure, which concur with previous studies [29]. However, the findings from this study are region-specific.

## Limitations

We were not able to obtain a known G145R escaped mutant to use as a control, and, thus, we relied on sequencing of the gene. The ability of the S region to secrete surface antigen was not determined in vitro, but carriers of this escaped mutant are being followed up.
